# Young people’s parental discussion about sexual and reproductive health issues and its associated factors in Awabel woreda, Northwest Ethiopia

**DOI:** 10.1186/s12978-016-0143-y

**Published:** 2016-03-08

**Authors:** Atitegeb Ayehu, Teketo Kassaw, Getachew Hailu

**Affiliations:** Students Clinic, Debre Markos University, PO Box: +251-269, Debre Markos, Ethiopia; Department of Public Health, College of Medicine and Health Sciences, Debre Markos University, PO Box: +251-269, Debre Markos, Ethiopia

**Keywords:** Sexual and reproductive health, Adolescent, Youth, Young people, Reproductive health, Parental communication

## Abstract

**Background:**

In Ethiopia besides the very low health seeking behavior of young people, they do not have access to sexual and reproductive health information and even the existing health services are adult-centered. Furthermore, health providers are not well equipped in addressing young people sexual and reproductive health needs. Therefore, parent-young people discussion about sexual and reproductive health issues are crucial in increasing their awareness and reduces their risky sexual behaviors. This study was aimed to assess young people’s parental discussion about sexual and reproductive health issues and its associated factors in Awabel woreda, Northwest Ethiopia.

**Methods:**

A community based cross-sectional study was conducted among 781 young people aged 10–24 years in Awabel Woreda, Northwest Ethiopia. A pre-tested structured interview administered questionnaire was used for the data collection. The collected data were entered using Epi Data 3.1 and analyzed using SPSS for windows version 21.

**Results:**

In the past 6 months, about one quarter, 25.3 % of young people had a parental discussion about sexual and reproductive health issues. Young people who reside in urban areas were more likely to discuss on sexual and reproductive health issues with their parents [AOR = 2.44, 95 % CI: 1.54–3.89]. Similarly, being male was more likely to have a parental discussion about sexual and reproductive health issues than females [AOR = 1.63, 95 % CI: 1.11–2.38]. Furthermore, the odds of parent-young people discussion about SRH matters was more likely among young people aged 20–24 years [AOR = 4.57, 95 % CI: 2.13–9.82], living with fathers [AOR = 2.46, 95 % CI: 1.20–5.04] and had attained a primary level of education [AOR = 2.89, 95 % CI: 1.22–6.87]. Parents lack of interest to discuss, feeling ashamed and culturally not acceptable to talk about sexual matters were found to deter young people’s in discussing sexual and reproductive health matters.

**Conclusion:**

Parent-young people discussion about sexual and reproductive health is very low and there are different hindering factors. And therefore, young people’s sexual and reproductive health programs or policies should be designed in addressing the cultural and societal factors besides the individual or behavioral factors.

## Background

There is overwhelming evidence on the importance of involving parents as part of the comprehensive strategy for improving young people’s health and development, however, there has been conflicting study’s findings on whether parents in Sub-Saharan Africa discuss with their children about sexual and reproductive health and on the effect of such discussion on young people’s sexual behaviour [1]. Though some studies showed that young people who discussed about SRH issues with their parents were less likely to engage in risky sexual behaviours [2, 3], but this finding was not consistent with other studies [4, 5]. A systematic review of behavioural studies found that family connectedness, and general and sexuality-specific parent-young people discussion had a protective association with young people sexual and reproductive health outcomes [6].

A qualitative study done in rural Tanzania on parent–child communication on sexual and reproductive health revealed that, though there were some communication; mainly focus on the same sex basis, about sexual and reproductive health issues in most families, the communication was characterized by parental warnings, threats and physical discipline. The topics of discussion were mainly on abstinence, unplanned pregnancy and HIV/AIDS, reflecting the worries that parents had on their children’s sexual health. The parent–child communication is limited by cultural barriers and the parents’ lack of knowledge about sexual and reproductive health issues [7]. Similarly, in Bangladesh the socio-cultural norms inhibit disclosure of information about sexual activities and other reproductive health issues for unmarried young people [8].

Young people in Ethiopia do not have access to information on sexual and reproductive health issues which have a great impact on their health [9, 10]. Besides the very low health seeking behavior of young people in Ethiopia, particularly sexual and reproductive health [11], the existing sexual and reproductive health services are adult-centered making them less accessible [12]. Moreover, health service providers are not well equipped in addressing young people’s specific health needs [13] and therefore, in this situation, parents, community members and other stakeholders is really crucial in improving the health status of the young people [14].

Even though most young people knew about sexually transmitted infections, particularly HIV/AIDS and contraceptives, they failed to discuss on sexual and reproductive health issues with their parents. More than half of them preferred to discuss about sexual and reproductive health topics with peers and it was due to their lack of communication skill and feeling being ashamed [15]. Due to lack of knowledge, sociocultural norms and parental fear that discussion about reproductive health issues would encourage premarital sex, parent-young people discussion about sexual and reproductive health issues was rare in the Eastern part of Ethiopia, Harar [16].

Parent-young people discussion about sexual and reproductive health issues is believed to be culturally shameful in Ethiopia [17]. Lack of proper knowledge, besides the attached socio-cultural taboos, about sexual and reproductive health matters makes the parent-young people SRH open discussions difficult. This difficulty can be judged from a study done in Zway, Ethiopia, where only 20 % of the parents ever had a discussion on SRH issues with their young people [18].

Though, the two studies conducted in the two regions of the country, Ethiopia showed that 32.5 % of young people in Oromia region [19] and 43.5 % of young people in Tigray region [20] had a parental discussion about sexual and reproductive health issues but the discussions were infrequent and took the form of warning and threatening way [19]. Age, residence, education and living arrangement of young people was significantly associated with parent-young people Sexual and Reproductive Health (SRH) discussion. Young people failed to discuss on sexual and reproductive health issues due to fear of parents, cultural taboos attached to sex, embarrassments, and parents’ lack of knowledge of sexual and reproductive health [19]. Furthermore, in Machakle woreda, Northwest Ethiopia three quarters of young people had never discussed on sexual and reproductive health issues with their parents due to its worthlessness, fear, social and cultural taboos attached to it [21].

However, the Ethiopian government had developed a national reproductive health strategy [22] but the role of parent-young people discussion on sexual and reproductive health issues and its current status is not well addressed yet. Parental discussion about sexual and reproductive health issues is very important in improving young people’s sexual and reproductive health knowledge, thereby increasing their access and utilization of the services. Therefore, later on, this will bring a reduction of the burden of young people’s disease and disabilities associated with sexual and reproductive health. Thus, the purpose of this study was to determine the level of parental discussion about sexual and reproductive health issues and to identify the factors associated with it among young people in Northwest Ethiopia.

## Methods

### Study setting and period

The study was conducted in Awabel Woreda (woreda is Ethiopian common name for district), Northwest Ethiopia from September 1, 2014 to June 30, 2015. The woreda is located at a distance of 259 km to the Northwest of Addis Ababa, in the Amhara Region. It has a total of 32,253 households and 138,687 populations; 64,506 of them are young people [23]. There are 37 health facilities; six health centers, 28 health posts and three private clinics; and a total of 108 health workers and 56 health extension workers in the woreda [24].

### Study design and sampling

It was a community based cross-sectional study conducted among young people in Awabel Woreda, Northwest Ethiopia. The sample size, i.e., 781 was determined using a single population proportion formula taking a proportion (p) of youth friendly reproductive health service utilization in Harar at 63.8 % [25], a 95 % confidence level, 5 % margin of error, a design effect of 2 and 10 % of non-response rate.

The study participants were selected using a multistage sampling technique: at stage one eight kebeles (kebele is the lowest administrative unit in Ethiopia) were selected from the total 29 kebeles; one urban and 28 rural kebeles, in the woreda. One urban kebele (Lumamie Town) was directly taken while seven rural kebeles (25 % of the 28 rural kebeles) were taken by using simple random sampling method. In the next stage, using simple random sampling technique, a total of 9216 households having young people were selected in each of the sampled kebeles. Finally 781 young people were selected using the existing Health Post Family Folder through the simple random sampling technique with proportionate allocation to size. Per each household, one eligible young person was interviewed and two visits were made for absences in the first visit.

### Data collection

A structured interview-administered pre-tested Amharic questionnaire was used for the data collection. The purpose of the study was briefly introduced for each of the study participants and data were collected after obtaining a verbal informed consent. The data were collected by trained ten data collectors with health background and two BSc public health supervisors. The data were collected in the quietest corner of young people’s house where there was no noise and disturbance. The data collection process had taken an average of 40 minutes.

Data quality was assured through careful questionnaire design, pretest and training. One day training about the purpose of the study, the questionnaire in detail, the data collection procedure, the data collection setting and the rights of study participants in detail was given for the data collectors and supervisors. After each day of data collection, the collected data were checked for completeness and consistency by holding a meeting with the data collectors.

### Data analysis

In this study the term ‘parent-young people SRH discussion’ refers to parents initiating a discussion on sexual and reproductive health issues, or parental involvement in sexual and reproductive health discussion initiated by the young people, or both. It was measured with a ‘Yes’ for those who had a parental discussion and ‘No’ option for those who had no parental discussion as mentioned above.

Data were entered using Epi Data version 3.1 and then exported to SPSS version 21.0 for analysis. Descriptive statistics was used to describe the study population in relation to relevant variables. Bivariate and multivariable logistic regression was done to assess any significant relationship between each independent variable (sociodemographic characteristics) and outcome variable (SRH parental discussion). Crude and adjusted odds ratios were used to ascertain any associations between the dependent and independent variables while significance was determined using a 95 % confidence interval. For not losing the most important variables like mother’s education, independent variables with a *p*-value of less than 0.20 at the bivariate level were included in a multivariable logistic regression model. However, any significant association was determined at a *p*-value of less than 0.05 in the multivariable logistic regression model to control potential confounding variables.

### Ethical consideration

Ethical approval was obtained from Debre Markos University, College of Medicine and Health Sciences, Research Ethics Committee and a letter of permission was obtained from the Awabel Woreda Health Office. The purpose of the study was explained to young people and a verbal informed consent was obtained from the participants. For those study participants who were under the age of consent, informed verbal assent was obtained from their parents. Confidentiality of information was maintained by omitting any personal identifier from the questionnaires.

## Results

### Socio-demographic characteristics

Out of the 781 randomly selected young people, 746 were participated obtaining a response rate of 95.5 %. Above half, 389 (52.1 %), of them were females and 545 (73.1 %) were rural residents. The mean age of them was 17.80 (±2.65) years and the majority of them, 438 (58.7 %) were in the age group of 15–19 years. Nearly one third, 240 (32.2 %), of young people had attained a preparatory level of education and 568 (76.1 %) were students in occupation. Concerning their marital status, 649 (87.0 %) were single and most, 471 (63.1 %) of them were living with both parents. Six hundred eleven (81.9 %) of their mothers and 373 (50.3 %) of fathers were illiterate and can read and write in their education respectively (see Table 1).

**Table 1:** Socio-demographic characteristics of young people in Awabel Woreda, Northwest Ethiopia, 2015

Characteristics of respondents (*n* = 746)		Numbers	Percent
Sex	Male	357	47.9
	Female	389	52.1
Age Group in Years	10–14	107	14.3
	15–19	438	58.7
	20–24	201	26.9
Religion	Orthodox	669	89.7
	Muslim	65	8.7
	Protestant	12	1.6
Residence	Rural	545	73.1
	Urban	201	26.9
Educational Status	Illiterate	50	6.7
	Read and Write Only	39	5.2
	Primary	204	27.3
	Secondary	213	28.6
	Preparatory	240	32.2
Marital Status	Single	649	87.0
	Married	97	13.0
Ethnicity	Amhara	739	99.1
	Oromo	7	0.9
Living Arrangement	Both parents	471	63.1
	Father only	112	15.0
	Mother only	66	8.8
	With couple	97	13.0
Mother’s Education	Illiterate	611	81.9
	Read & Write Only	75	10.1
	Primary	57	7.6
	Secondary and Above	3	0.4
Father’s Education	Illiterate	278	37.3
	Read & Write Only	375	50.3
	Primary	59	7.9
	Secondary and Above	34	4.6
Occupational Status	Housewife	74	9.9
	Daily Laborer	100	13.4
	Student	572	76.7

### Young people’s parental discussion about sexual and reproductive health issues

**Fig. 1:**
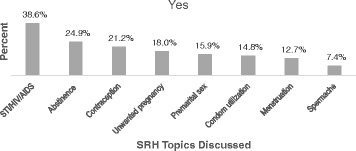
Sexual and reproductive health topics discussed among young people with their parents in Awabel Woreda, Northwest Ethiopia, 2015 (*n* = 189)

Above half of young people, 386 (51.7 %) had accepted that it is important to discuss about sexual and reproductive health issues with their parents. However; only 189 (25.3 %) of them had a parental discussion on at least one sexual and reproductive health issues in the past 6 months. Out of the 189 young people who had a parental discussion, a very low proportion of them had discussed with their parents about biological aspects of sexual and reproductive health issues such as spermache, 14 (7.4 %) and menstruation, 24 (12.7 %). Forty seven (24.9 %) and 40 (21.2 %) of young people had reported discussion about the preventive aspects like: abstinence and contraception respectively. Furthermore, 73 (38.6 %) about STI/HIV/AIDS and 34 (18.0 %) of them had discussed about unwanted pregnancy (See Fig. 1).

**Fig. 2:**
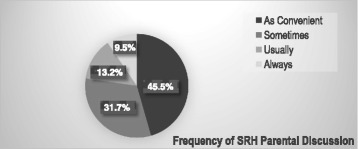
Frequency of parental discussion on sexual and reproductive health issues among young people in Awabel Woreda, Northwest Ethiopia, 2015 (*n* = 189)

Individuals involved in the discussion about sexual and reproductive health issues were mothers, 59 (31.4 %); sisters, 54 (28.7 %); fathers, 52 (27.7 %) and brothers, 23 (12.2 %). Most; 86 (45.5 %) of the discussions were made as it was convenient followed by sometimes, 60 (31.7 %) (See Fig. 2). On the other hand, 431 (57.8 %) of them had SRH discussion other than parents, such as with peers, 187 (43.4 %); teachers, 106 (24.6 %); health providers, 99 (23.0 %) and peers’ parents, 39 (9.0 %).

**Fig. 3:**
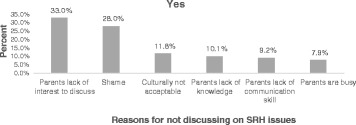
Reasons of young people for not discussion on SRH issues with their parents in Awabel woreda, Northwest Ethiopia, 2015 (*n* = 557)

The young people’s reason for not discussing about sexual and reproductive health issues with parents are shown in Fig. 3. The majority of the reasons were parents’ lack of interest to discuss or not a good listener, 184 (33.0 %) and fear of parents/shame, 156 (28.0 %) (See Fig. 3).

### Factors associated with young people’s parental discussion about SRH issues

In the bivariate analysis sex, residence, age, living arrangement, educational attainment, and father’s education showed significant association on parental discussion about sexual and reproductive health issues. However, in the multivariable analysis father’s and mother’s education didn’t show any significant association with a parental discussion about SRH issues.

The odds of discussing on sexual and reproductive health issues were 1.63 times more likely in males compared to females’ [Adjusted Odds Ratio (AOR) = 1.63, 95 % CI: 1.11 - 2.38]. Young people within the age of 20–24 years were 4.57 times more likely to discuss on SRH issues with their parents than those within the age of 10–14 years [AOR = 4.57, 95 % CI: 2.13 - 9.82]. Similarly, the odds of discussing about sexual and reproductive health were 2.46 times higher among young people who were living with their fathers than those living with their couple [AOR = 2.46, 95 % CI: 1.20 - 5.04]. Those respondents who had attained primary level of education were 2.89 times more likely to have odds of communication about sexual and reproductive health issues than those who can’t read and write [AOR = 2.89, 95 % CI: 1.22 - 6.87]. Furthermore, the odds of SRH parental discussion were 2.44 times more likely among young people who reside in urban areas than those living in rural areas [AOR = 2.44, 95 % CI: 1.54 - 3.89] (See Table 2).

**Table 2:** Factors associated with young people’s parental discussion on sexual and reproductive health issues in Awabel Woreda, Northwest Ethiopia, 2015

Variables (*n* = 746)		SRH parental discussion		Crude odds ratio (95 % C.I.)	Adjusted odds ratio (95 % C.I.)
		Yes n (%)	No n (%)		
Sex	Female	123 (31.6)	266 (68.4)	1.00	1.00
	Male	66 (18.5)	291 (81.5)	2.04 (1.45 – 2.87)***	1.63 (1.11 – 2.38)*
Residence	Rural	157 (28.8)	388 (71.2)	1.00	1.00
	Urban	32 (15.9)	169 (84.1)	2.14 (1.40 – 3.26)***	2.44 (1.54, 3.89)***
Age Group in Years	10–14	21 (19.6)	86 (80.4)	1.00	1.00
	15–19	148 (33.8)	290 (66.2)	0.48 (0.29 – 0.80)**	0.97 (0.53 – 1.77)
	20–24	20 (10.0)	181 (90.0)	2.21 (1.14 – 4.29)*	4.57 (2.13 – 9.82)***
Living Arrangement	Couple	31 (32.0)	66 (68.0)	1.00	1.00
	Both Parents	119 (25.3)	352 (74.7)	1.39 (0.86 – 2.23)	1.60 (0.93 – 2.76)
	Father only	20 (17.9)	92 (82.1)	2.16 (1.13 – 4.12)*	2.46 (1.20 – 5.04)*
	Mother only	19 (28.8)	47 (71.2)	1.16 (0.59 – 2.30)	1.16 (0.53- 2.56)
Educational Status	Illiterate	11 (22.0)	39 (78.0)	1.00	1.00
	Read and Write	7 (17.9)	32 (82.1)	1.29 (0.45 – 3.71)	1.61 (0.53 – 4.88)
	Primary	20 (9.8)	184 (90.2)	2.60 (1.15 – 5.85)*	2.89 (1.22 – 6.87)*
	Secondary	79 (37.1)	134 (62.9)	0.48 (0.23 – 0.99)*	0.51 (0.23 – 1.13)
	Preparatory	72 (30.0)	168 (70.0)	0.66 (0.32 – 1.36)	0.45 (0.20 – 1.01)
Mother’s Education	Illiterate	152 (24.9)	459 (75.1)	1.00	1.00
	Read and write only	22 (29.3)	53 (70.7)	0.80 (0.47 – 1.36)	0.73 (0.40 – 1.34)
	Primary	13 (22.8)	44 (77.2)	1.12 (0.59 – 2.14)	0.97 (0.48 – 1.99)
	Secondary and above	2 (66.7)	1 (33.3)	**0.17 (0.02 – 1.84)** ^**+**^	0.34 (0.03 – 4.17)
Father’s Education	Illiterate	65 (23.4)	213 (76.6)	1.00	1.00
	Read and write only	96 (25.6)	278 (74.4)	0.89 (0.62 – 1.27)	1.06 (0.71 – 1.59)
	Primary	32 (37.3)	37 (62.7)	0.51 (0.28 – 0.93)*	0.65 (0.34 – 1.26)
	Secondary and above	6 (17.6)	28 (82.4)	1.42 (0.57 – 3.59)	2.10 (0.78 – 5.68)

Significant at *p-value < 0.05, **p-value < 0.01, and ***p-value < 0.001, whereas ^+^ *p*-value < 0.20

## Discussion

This study revealed that about one quarter of the young people had a parental discussion about sexual and reproductive health issues in Awabel woreda. The discussion was significantly higher, especially among young males and young adult, urban residents, lived with fathers, and had a primary level of educational attainment.

In the past 6 months young people’s parental discussion about sexual and reproductive health issues was low (25.3 %). Though the method of data collection and study participants (parents) differs, the finding was comparable (26 %) with a study done in the United States of America [26]. This finding was a bit higher than a study conducted in Lesotho (20 %) [27], but, lower than other studies done in Ethiopia, i.e., Debre Markos Town [28] and Bulen Woreda [29] and studies conducted in Ghana (82.3 %) [30], Zimbabwe (44 %) [31] and Mexico (83.1 %) [32]. This difference might be due to differences in the study population, study settings, data collection method and the study design itself as most of the above mentioned studies were conducted among students and urban residents. Furthermore, it might be due to demographic and cultural differences and difference in accessing sexual and reproductive health information.

Parental discussion about sexual and reproductive health issues were significantly associated and higher among young males. This might be due to the fact that males might not be influenced by the social taboos than females. Similarly, systematic review of literatures in Latin America and Caribbean countries found that parent-young people discussion about sexual and reproductive health matters seemed to be more protective for females than males [33]. A qualitative study done in Tanzania revealed that parent-young people discussion about SRH issues was mainly on the same sex basis [7]. This was indicated by other studies done in Bulen Woreda [29] and China [34] as there was a significant gender difference in the pattern of parental discussion about sexual and reproductive health issues. Furthermore, males might have a better educational attainment than females enhancing their knowledge and motored them to discuss about sexual and reproductive health matters.

Young people who reside in urban areas were more likely to discuss on sexual and reproductive health issues than rural residents. This finding was supported by a study done in Eastern Wollega [19]. As studied in Tanzania, young people were hesitated from discussing SRH topics with their parents due to lack of trust and fear of punishment. There in Tanzania, parents were also limited to discuss about SRH issues with their child due to the cultural norms that restricted interactions between opposite sex and lack of appropriate knowledge about SRH matters [7]. However, the aforementioned issues might not be as such a major challenge in urban areas as urban young people might have better knowledge, access to information or media exposure to SRH issues as compared to rural residents and their parents might be educated. This was supported by a study done in Debre Markos town, Ethiopia as young people who had SRH information were more likely to discuss about SRH issues with their parents than those who didn’t get SRH information [28].

This study showed that parental discussion about sexual and reproductive health issues was significantly associated and higher with an increase in respondent’s age and it was in agreement with other study done in Nekemte, Ethiopia [19] and Nigeria [35]. This could be due to the better understanding of sexual and reproductive health issues influencing them to discuss on it. Earlier studies have also indicated that the extent of communication on sexual and reproductive health matters increases with age and continuing through young adulthood [36].

Living arrangement was significantly associated and higher among young people who were living with their fathers to discuss on SRH matters. Whatever the health services used or the discussions made, living arrangement has influences on sexual and reproductive health issues [37]. This finding was not supported by a study done in Eastern Wollega as young people who were living with other relatives were more likely to discuss on SRH topics [19]. This could be related to their fathers’ educational status, and even these young people might be educated and lived in urban areas. Moreover, this could be influenced by gender difference in the pattern of parental discussion about sexual and reproductive health issues as revealed by other studies done in Ethiopia [29] and China [34].

Young people’s primary level of educational attainment was significantly associated and higher to discuss on SRH issues as compared to those who were illiterate. This finding was supported by a study done in Eastern Wollega [19] and Nigeria [35] as their educational attainment increased they were more likely to discuss about sexual and reproductive health issues. However, it was in contrast to a study done in Debre Markos town [28] as young people with a grade level of the 12^th^ were less likely to discuss about SRH topics. In this study young people might overcome the social taboos and their parents might give time to discuss about sexual and reproductive health matters. In addition, age of young people might have influence on sexual and reproductive health discussion, especially for those in the later ages of young people.

Since this study is a community based study done among urban and rural young people and explored the different independent variables, this gives a better and balanced picture of the situation. Moreover, this study used the recent information (6 months) to minimize recall bias. However, this study has its own limitation in that the participants’ response might have been affected by social desirability which might affect the validity of the result. The fact that the design was cross sectional, may hinder the determination of causality of the relationship. In addition, the in-depth reasons why young people didn’t discuss about sexual and reproductive health issue with their parents were not explored through a qualitative study.

This study has a good implication for clinicians in addressing parent-young people communication about sexual and reproductive health issues through providing age appropriate educational health services (sexuality education) for all young people to help them develop communication skills and responsible sexual behaviors. It has also a good implication for policy makers to revitalize the existing national reproductive health strategy. In addition, it gives a good insight for researchers to explore the societal structure in relation to parent-young people sexual and reproductive health discussion. Furthermore, it implies the need for sustainable advocacy works targeting parents and communities on young people’s sexual and reproductive health communication.

This study did not address the societal structures how the issues of sexual and reproductive health discussed in the study area. Since it is important to have a comprehensive community based data and the barriers related to parent-young people sexual and reproductive health communication, in the future, ethnographic studies should be conducted to explore the Ethiopian societal structure in relation to parent-young people discussion about sexual and reproductive health issues.

## Conclusion

This study showed that young people’s parental discussion about sexual and reproductive health issues was low in Awabel Woreda. Parental discussion was significantly higher among young males and young adults, who lived in urban areas and with their father only and had a primary level of educational attainment. And therefore, young people’s sexual and reproductive health programs or policies should be designed in addressing the cultural and societal factors besides the individual or behavioral factors.
